# Hippocampal subfield plasticity is associated with improved spatial memory

**DOI:** 10.1038/s42003-024-05949-5

**Published:** 2024-03-05

**Authors:** Henning Boecker, Marcel Daamen, Lukas Kunz, Melanie Geiß, Moritz Müller, Thomas Neuss, Leonie Henschel, Rüdiger Stirnberg, Neeraj Upadhyay, Lukas Scheef, Jason A. Martin, Tony Stöcker, Alexander Radbruch, Ulrike Attenberger, Nikolai Axmacher, Angelika Maurer

**Affiliations:** 1 Clinical Functional Imaging Lab, Department of Diagnostic and Interventional Radiology, University Hospital Bonn, Venusberg-Campus 1, 53127 Bonn, Germany; 2 German Center for Neurodegenerative Diseases, Venusberg-Campus 1/99, 53127 Bonn, Germany; 3 Department of Epileptology, University Hospital Bonn, Venusberg-Campus 1, 53127 Bonn, Germany; 4 Department of Neuroradiology, University Hospital Bonn, Venusberg-Campus 1, 53127 Bonn, Germany; 5 Department of Diagnostic and Interventional Radiology, University Hospital Bonn, Venusberg-Campus 1, 53127 Bonn, Germany; 6 Department of Neuropsychology, Faculty of Psychology, Ruhr University Bochum, Universitätsstr. 150, 44801 Bochum, Germany

**Keywords:** Hippocampus, Cognitive ageing

## Abstract

Physical exercise studies are generally underrepresented in young adulthood. Seventeen subjects were randomized into an intervention group (24.2 ± 3.9 years; 3 trainings/week) and 10 subjects into a passive control group (23.7 ± 4.2 years), over a duration of 6 months. Every two months, performance diagnostics, computerized spatial memory tests, and 3 Tesla magnetic resonance imaging were conducted. Here we find that the intervention group, compared to controls, showed increased cardiorespiratory fitness, spatial memory performance and subregional hippocampal volumes over time. Time-by-condition interactions occurred in right cornu ammonis 4 body and (trend only) dentate gyrus, left hippocampal tail and left subiculum. Increases in spatial memory performance correlated with hippocampal body volume changes and, subregionally, with left subicular volume changes. In conclusion, findings support earlier reports of exercise-induced subregional hippocampal volume changes. Such exercise-related plasticity may not only be of interest for young adults with clinical disorders of hippocampal function, but also for sedentary normal cohorts.

## Abstract (web summary)

A structural MRI study suggests that regular exercise training induces plasticity in hippocampal subregions also in young adults. Hippocampal plasticity correlates with changes in spatial memory precision.

## Introduction

The key role of the human hippocampus for higher mnemonic functions, e.g., episodic and visuospatial memory^1^, is long-established, as well as contributions of hippocampal dysfunction to memory impairments in various clinical disorders (e.g., dementia, major depression)^2,3^: accordingly, there is a growing interest in pharmacological and non-pharmacological treatments which could help to improve hippocampal function. Physical activity as a low-cost low-risk intervention provides an attractive option, across various clinical populations and age groups^4^. Human magnetic resonance imaging (MRI) studies provided firm indications that regular exercise is associated with higher structural integrity of the hippocampus, including meta-analytical evidence^4–8^, but only few studies were designed as randomized controlled trials (RCTs) comparing physical activity and control interventions^9–14^. A recent meta-analysis on global hippocampal volume concluded that exercise-induced plasticity was evident in older^9,11,12^, but not young adults^6^. Meanwhile, RCT studies in young to middle-aged adults are rare, and often used very short training periods of only a few weeks^15–18^. Global hippocampal volume measures may also not be sensitive enough in this age range, where neither childhood and adolescent growth nor age-related atrophy drive the developmental trajectory. Therefore, a main goal of this study was to examine whether subregional hippocampal volumes provide more sensitive measures of exercise-induced plasticity in this age range after a longer training duration of 6 months.

Specifically, analyses of total hippocampal volume may miss selective effects in hippocampal subregions. Growing evidence suggests functional gradients along the hippocampal longitudinal axis, as reflected by differential anatomical circuitries of anterior, body, and posterior subregions^19–21^. Some data indicate fitness-related changes preferentially in anterior (head) subregions of the hippocampus^9,11,18,22,23^. Moreover, the hippocampus proper consists of several subfields with varying cytoarchitecture, including the cornu ammonis areas (CA1/CA2/CA3/CA4), dentate gyrus and subiculum. Here, the dentate gyrus is of special interest: In rodents, physical activity induces hippocampal cell proliferation, neurogenesis, and synaptic plasticity almost exclusively in this area, and this process is also associated with improved spatial memory performance^24–26^, though it may also foster forgetting of preexisting memories^27,28^. Although difficult to measure in vivo, indirect evidence (e.g., for angiogenesis^29^) suggests that physical activity also promotes neurogenesis in the human hippocampus^30^. While cross-sectional studies observed positive correlations between aerobic fitness level and larger CA1 volumes in male adolescents^31^, and larger volumes for bilateral CA1, CA4, dentate gyrus granule cell and molecular layers, left CA2–3, and the left hippocampal amygdaloid transition area in middle-aged amateur marathon runners^32^, complementary evidence from RCT studies in young adulthood is limited and mixed: In addition to CA4/dentate gyrus volume increases after a 16-weeks training^16^ and positive associations between cardiorespiratory fitness improvements and dentate gyrus/CA3 head volume changes after a 12-weeks training^23^, an earlier study also suggested CA2/3, subiculum and dentate gyrus volume decreases after 6-weeks intense aerobic exercise training^18^.

Varying effects of physical exercise on hippocampal subfields may also have differential impact on cognitive functions that depend on the hippocampus and surrounding medial temporal regions, including episodic and spatial memory^1^. During long-term memory consolidation, the hippocampus is thought to bind together afferent item memory (e.g., objects) and context memory (e.g., spatial locations) information from the perirhinal and parahippocampal cortex, respectively, which is relayed via the entorhinal cortex^33–35^. This relational memory is crucial, for instance, for remembering the precise location of objects in space, which requires information about objects to be integrated into a spatial framework. Within the hippocampus, dentate gyrus granule cells support pattern separation by transforming rich cortical inputs via the entorhinal cortex into sparse outputs towards pyramidal cells in CA3, and the hippocampal output region CA1^36–38^. Pattern separation (i.e., computational processes that assure that similar memory contents are stored and retrieved as distinct memory representations) is believed to be a hallmark feature of episodic memory, but also spatial mnemonic processing^35,37–39^.

Various behavioral paradigms aim to maximize demand for this kind of mnemonic discrimination, also in exercise-related studies. For example, a cross-sectional study in young adults^40^ observed that higher cardiorespiratory fitness was linked with better Mnenomic Similarity Task performance: in this object recognition paradigm, participants have to disambiguate repetitions of original stimuli from lure stimuli which look similar, but not identical to the originals^41^, and better performance is often interpreted as indirect evidence for better dentate gyrus/CA3 function. Cardiorespiratory fitness increases after a 12-weeks training correlated with improved performance in a facial memory paradigm, at least for participants with low baseline fitness^23^. Meanwhile, some studies cannot replicate such associations^42^, or only in elderly^43^. Notably, one cross-sectional study^44^ found no associations between physical fitness and performance in the original task, but reported a fitness-related attenuation of age-related decline in a virtual 3D navigation task that afforded mnemonic discrimination of overlapping routes.

This could indicate a higher sensitivity of tasks with stronger spatial (instead of purely object-related) demands, which concurs with observations from similar paradigms. A seminal RCT^9^ observed that exercise-related increases of hippocampal volume in older adults were correlated with improved performance in a delayed matching-to-sample task where participants had to remember the precise spatial location of dot stimuli on a 2D display. Moreover, a cross-sectional study^45^ in young adults observed that higher cardiorespiratory fitness was associated with smaller placement errors in a paradigm where young participants had to actively reconstruct the 2D spatial arrangement of five abstract shapes that were displayed immediately before, suggesting higher spatial memory precision. Interestingly, this relationship was mediated by fitness-related differences in hippocampal MR viscoelasticity (but not volume) measures, and later work in young adults suggested a more specific relationship between CA3/dentate gyrus viscoelasticity and task performance^46^. Meanwhile, hippocampal and surrounding parahippocampal areas are involved in 3D spatial navigation^47^, e.g., in variants of the Morris water maze^48^, where the position of a hidden location inside a circular arena has to be recollected by triangulating it from the positions of landmarks outside the arena. Preliminary human data using virtual variants of this task suggest that efficient performance is associated with hippocampal volume^49,50^, and CA1/2 volumes more specifically^51,52^. Moreover, cross-sectional studies in adolescents observed that higher physical fitness levels are linked with better task performance and higher hippocampal^53^ and CA1 subfield^31^ volumes, but found no evidence for a mediation. Therefore, while some cross-sectional data in young adults suggest positive associations between spatial memory performance and physical fitness, whether fitness increases are linked with improved spatial memory performance still needs to be clarified.

**Fig. 1:**
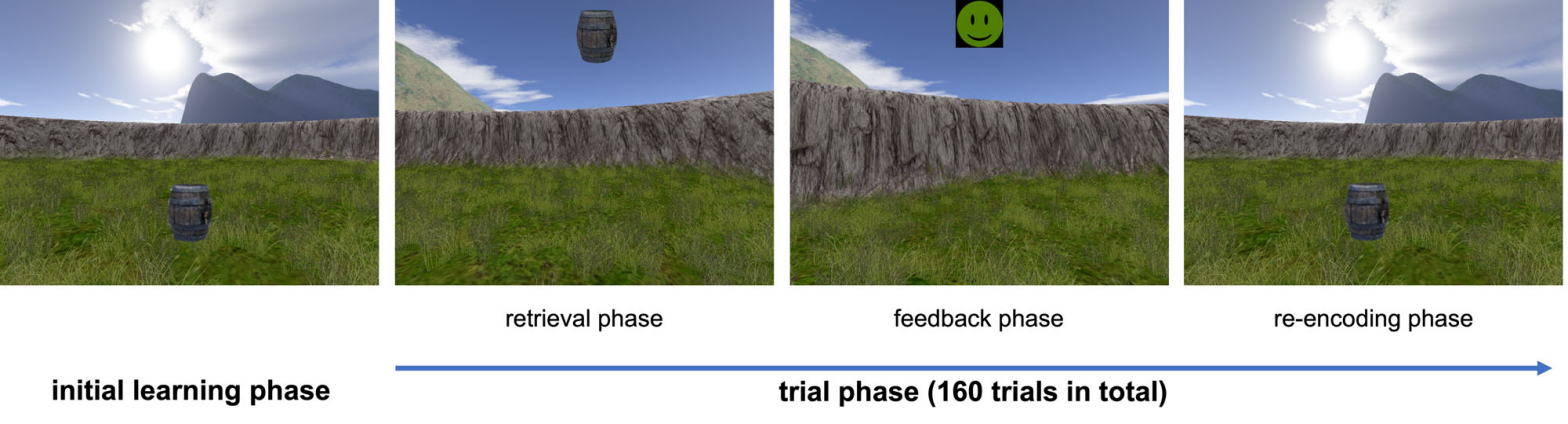
Spatial object memory task^56,77^. Participants had to perform a computerized object-location memory task while navigating freely in a circular virtual arena: After an initial learning presentation of an everyday object in a specific spatial position, during subsequent retrieval, participants had to navigate through the arena, and place the cued object as accurately as possible at its original location. Participants received feedback about their accuracy via smiley faces, followed by a re-encoding of the correct object position, to facilitate incremental learning.

Given the sparsity of data addressing exercise-induced hippocampal subregional plasticity in young adults, the current RCT explored the effects of a 6-months aerobic exercise intervention on hippocampal subregional plasticity and its specific relation to spatial object memory in sedentary young adults. We examined an intervention group (INT) that received regular exercise training and a no-training control group (CON) who both performed MRI and cognitive testing at baseline (T0m), and 2-months (T2m), 4-months (T4m), and 6-months (T6m) follow-ups. We hypothesized to observe differential volume changes in hippocampal subregions, as derived from high-resolution structural MRI, with a special theoretical focus on the dentate gyrus^54^. Moreover, we tested whether subfield-specific hippocampal changes were linked with training-induced improvements of spatial memory precision in a virtual arena paradigm (spatial object memory task^55,56^: Fig. 1), hence, supporting subregional hippocampal plasticity as a driver of spatial memory also in young adults.

Consistent with earlier studies in young adults, no training-related change of global hippocampal volume was observed, but circumscribed changes in specific subregions, suggesting that regular exercise can induce hippocampal plasticity in young sedentary adults. Both groups tended to show improved precision of spatial learning over the 6-months period, but this change was more robust for the INT group. Volume increases of the hippocampal body as well as, at subregional level, the left subiculum volume correlated with better spatial memory over the 6-months period, supporting structural-functional relationships between hippocampal plasticity and spatial memory changes. Yet, these associations were only robust across the whole sample, not within the exercise group only. Therefore, further evidence is needed to confirm the impact of training-induced hippocampal plasticity on complex spatial memory abilities in young adults.

## Results

### Participants

The cohort was already introduced in previous reports^57,58^. In total, *N* = 27 INT and *N* = 15 CON participants received their allocated treatment. Fourteen participants dropped out within the first 4 months or had to be excluded from final analyses for various reasons (including: loss of interest, time constraints, depressive symptoms, injury due to leisure activities, insufficient training participation due to illness, and non-eligibility for MRI). Further, one subject needed to be excluded from the present analyses due to lack of compliance in the Spatial Object Memory Task. This resulted in a final sample size of *N* = 27 participants: *N* = 17 in the INT and *N* = 10 in the CON group. For T6m follow-ups, MRI was missing in one participant due to new contraindications, and one participant performed neither MRI nor memory examinations, leaving data from *N* = 25 participants (INT: *N* = 15, CON: *N* = 10) for respective analyses. There was no significant difference in drop-out rates between INT and CON (*χ*^2^(1,42) = 0.058, *p* = 0.810), and no systematic difference in background characteristics between study drop-outs and completers (Supplementary Table 1).

Baseline sample characteristics are summarized in Table 1. In general, the sample included young adults who, on average, showed a normal body mass index (BMI) and a fair level of fitness, according to established criteria^59^ for maximum oxygen consumption relative to body weight (relVO_2max_). Independent *t*-tests and *χ*^2^ tests revealed no significant baseline group differences for the basic demographic, anthropometric, or psychiatric variables (e.g., age, BMI, relVO_2max_).

**Table 1:** Baseline sample characteristics

Variable	Intervention group mean ± standard deviation or *N*	Control group mean ± standard deviation or *N*	*p*-Values
Age [years]	24.2 ± 3.9	23.7 ± 4.2	0.769
Sex (male/female)	6/11	6/4	0.212
Height [cm]	172.8 ± 12.0	176.9 ± 7.9	0.341
Weight [kg]	67.1 ± 9.7	71.2 ± 14.1	0.372
BMI [kg∙m^-2^]	22.5 ± 2.6	22.7 ± 3.6	0.886
HR_max_ [bpm]	197.8 ± 7.1	200.8 ± 8.5	0.329
relVO_2max_ [ml min^−1^ kg^−^^1^]	38.7 ± 3.5	41.7 ± 7.5	0.258
Education [years]	16.4 ± 3.1	15.8 ± 3.1	0.655
WST IQ	106.8 ± 10.2	107.3 ± 8.8	0.903
EHI	73.9 ± 16.7	79.5 ± 13.3	0.374
BDI	2.1 ± 2.7	1.4 ± 1.5	0.484
STAI trait	32.8 ± 8.2	31.1 ± 5.8	0.663

Demographic, anthropometric and psychiatric background variables for the intervention group (*N* = 17) and control group (*N* = 10). Group differences tested with two-sided independent t-tests, except for sex (*χ*^2^ test). Abbreviations: *BDI* Beck Depression Inventory (0–10 points: no depression), *BMI* body mass index, *HR*_*max*_ maximum heart rate in performance diagnostic, *EHI* Edinburgh Handedness-Inventory—Laterality Quotient (value > 40 indicates right-handedness), *relVO*_*2max*_ maximum oxygen consumption relative to body weight, *STAI trait* trait anxiety of the State-Trait Anxiety Inventory (clinically relevant anxiety scores are ≥39), *WST IQ* verbal intelligence quotient derived from German vocabulary test.

### Physiological data—relVO_2max_

INT participants performed 60.1 ± 11.4 (77 ± 14%) trainings on average out of maximally 78 planned. Due to artifacts, relVO_2max_ values of two INT participants could not be determined, and were therefore excluded from analyses including relVO_2max_ data.

Data showed an increase in relVO_2max_ in INT and a slight decrease in CON participants (Table 2). The Linear Mixed Effect (LME) model for relVO_2max_ revealed a significant effect of time (*F*(3,64.18) = 5.26, *p* = 0.003) and time-by-group interaction (*F*(3,64.18) = 14.02, *p* < 0.001), however, no group effect (*F*(1,21.04) = 2.60, *p* = 0.122). Moreover, a significant effect of sex (*F*(1,29.99) = 23.22, *p* < 0.001) indicated higher relVO_2max_ values in males than females. Age had no significant impact (*F*(1,20.94) = 0.34, *p* = 0.566).

**Table 2:** Values of relative VO_2max_ across time points

	T0m mean ± standard deviation	T2m mean ± standard deviation	T4m mean ± standard deviation	T6m mean ± standard deviation
Intervention group	38.7 ± 3.5	41.3 ± 4.1	42.9 ± 5.6	42.6 ± 5.0
Control group	41.7 ± 7.5	39.1 ± 7.2	41.1 ± 8.4	40.3 ± 7.4

Maximum oxygen consumption relative to body weight [relVO_2max_, in ml min^−1^ kg^−1^] across time points, for the intervention group (*N* = 15) and control group (*N* = 10). Abbreviations: *T0m* baseline measurement, *T2m* measurement after 2 months, *T4m* measurement after 4 months, *T6m* measurement after 6 months.

Post hoc tests within the INT group showed a significant increase in relVO_2max_ from T0m to T2m (*t*(14) = 5.34, *p* = 0.001, *d* = 1.38), T0m to T4m (*t*(14) = 6.10, *p* < 0.001, *d* = 1.58), and T0m to T6m (*t*(13) = −5.86, *p* < 0.001, *d* = −1.57). For CON participants, no significant changes were detected (T0m to T2m: *t*(6) = −1.45, *p* = 1.000, *d* = −0.55; T0m to T4m: *t*(8) = −1.86, *p* = 0.601, *d* = −0.62; T0m to T6m: *t*(9) = 2.26, *p* = 0.303, *d* = 0.71). Comparing INT with CON revealed no significant differences at T0m (*t*(11.6) = −1.19, *p* = 0.258, *d* = −0.56), T2m (*t*(7.85) = 0.75, *p* = 0.474, *d* = 0.42), T4m (*t*(22) = 0.64, *p* = 0.528, *d* = 0.27), or T6m (t(22) = 0.92, *p* = 0.367, *d* = 0.38). Comparisons between groups for the deltas were all significant, showing an increase in fitness (relVO_2max_) in INT and decrease in CON participants (ΔT2T0: *t*(20) = 4.18, *p* < 0.001, *d* = 1.91; ΔT4T0: *t*(22) = 5.26, *p* < 0.001, *d* = 2.22; and ΔT6T0: *t*(22) = 5.61, *p* < 0.001, *d* = 2.52).

### Spatial object memory task—mean drop error

**Fig. 2:**
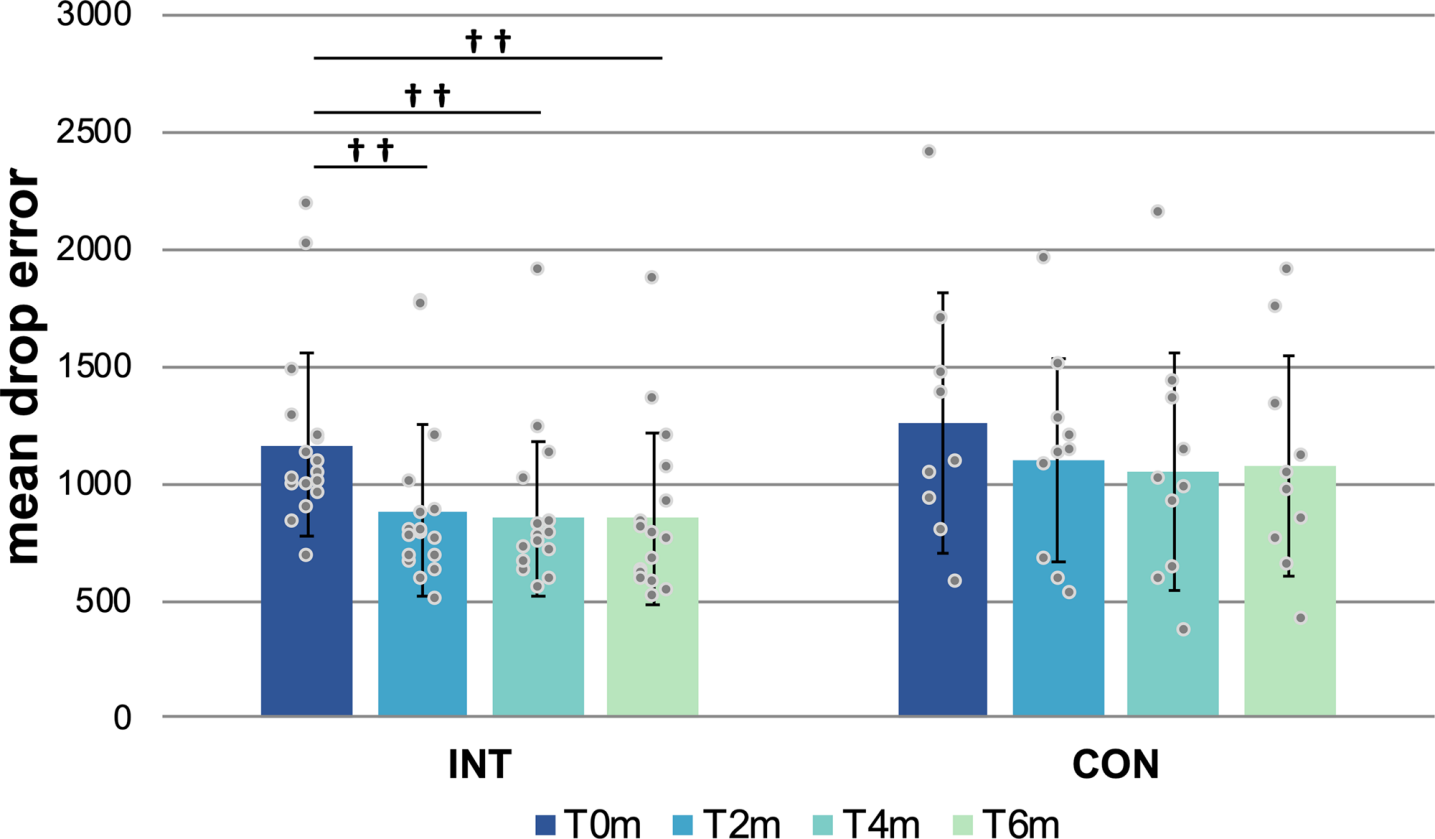
Mean drop error (virtual units) within groups (INT and CON) over all time points (T0m, T2m, T4m, and T6m). Raw values presented with means and standard deviations (as error bars); †† indicates within-group significances at *p* < 0.01. Data from *N* = 17 INT and *N* = 10 CON participants. Abbreviations: CON control group, INT intervention group, T0m baseline measurement, T2m measurement after 2 months, T4m measurement after 4 months, T6m measurement after 6 months.

The mean drop error (MDE) between the recalled and the actual spatial position of objects within the virtual arena decreased over time (Fig. 2), resulting in a significant main effect of time (*F*(3,73.10) = 13.58, *p* < 0.001), while no other significant effects were found (group: *F*(1,23.06) = 1.69, *p* = 0.207; time-by-group interaction: *F*(3,73.10) = 0.99, *p* = 0.403; age: *F*(1,23.00) = 0.01, *p* = 0.934; sex: *F*(1,23.06) = 0.56, *p* = 0.463). This indicates that precision of spatial memory performance generally improved across subjects over follow-ups.

Exploratory post hoc tests within the INT group showed significant MDE decreases from T0m and T2m (*t*(16) = −4.83, *p* = 0.001, *d* = −1.17), T0m and T4m (*t*(16) = −4.69, *p* = 0.001, *d* = −1.14) and T0m and T6m (*t*(15) = −4.49, *p* = 0.003, *d* = −1.12, whereas CON did not show any significant effects between time points, and smaller effect sizes (T0m to T2m: (*t*(8) = −2.59, *p* = 0.193, *d* = −0.86; T0m and T4m: (*t*(8) = −2.82, *p* = 0.136, *d* = −0.94; T0m and T6m: (*t*(8) = −1.47, *p* = 1.000, *d* = −0.49). Further, there were no significant effects between groups (neither between time points separately (T0m, T2m, T4m, or T6m) nor between the deltas (ΔT6T0, ΔT4T0, and ΔT2T0)).

When we investigated MDE in only the first 20 trials per session (motivated by the idea that these early trials might be more sensitive to physical activity-dependent changes), we obtained similar results as with all 160 trials per session (significant main effect of time: *F*(3,73.31) = 16.66, *p* < 0.001; no effect of group: *F*(1,23.14) = 1.19, *p* = 0.29; no time-by-group interaction: *F*(3,73.31) = 0.64, *p* = 0.59). Secondary analyses showed that participants became faster at completing the 160 trials per session over time (*F*(3,73.14) = 8.47, *p* < 0.001) and that INT and CON needed similar amounts of time to complete each session (*F*(1,23.01) = 3.50, *p* = 0.07).

### Structural data—whole hippocampus

Whole Hippocampus: Only a trend-level main effect of group (*F*(1,22.00) = 4.1, *p* = 0.055), but no main effect of time (*F*(3,73.00) = 0.7, *p* = 0.533) or time-by-group interaction (*F*(3,73.00) = 0.1, *p* = .960) were observed.

### Structural data—head/body/tail

For the head region, only a significant main effect of group (*F*(1,22.00) = 5.5, *p* = 0.029), but no main effect of time (*F*(3,73.01) = 0.4, *p* = 0.764) or time-by-group interaction (*F*(3,73.01) = 0.9, *p* = 0.437) were observed. Neither significant main (group: *F*(1,22.00) = 1.1, *p* = 0.312; time: *F*(3,73.00) = 0.5, *p* = 0.680) nor interaction effects (*F*(3,73.007) = 1.3, *p* = 0.275) were observed for the tail region.

Meanwhile, a significant interaction effect emerged in the hippocampal body (*F*(3,73.00) = 2.89; *p* = 0.041). No main effects (group: *F*(1,22.00) = 1.8, *p* = 0.190; time: *F*(3,73.0) = 0.5, *p* = 0.679) were observed.

**Fig. 3:**
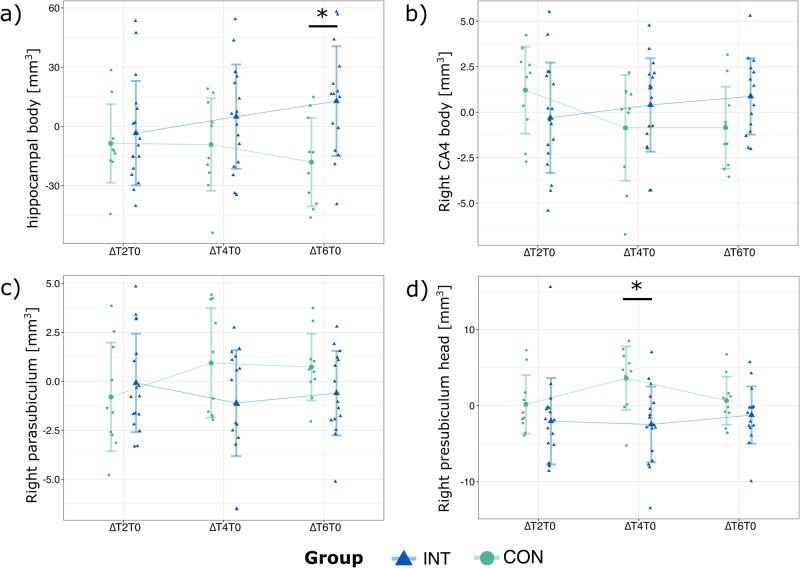
Hippocampal subfield volumes with significant time-by-group interactions. **a** Hippocampal body; **b** right CA4 body; **c** right parasubiculum, and **d** right presubiculum head. Presented are the deltas between T0m and T2m (ΔT2T0), T0m and T4m (ΔT4T0) as well as T0m and T6m (ΔT6T0) for both groups, along with means and standard deviations (as error bars). Data from *N* = 17 intervention (INT) and *N* = 10 control (CON) participants. * indicates between-group significances at *p* < 0.05.

Post hoc analyses revealed no significant effects between time points within the INT or CON. However, between-group comparisons revealed a significant difference for the change in volume ΔT6T0 (*t*(23) = 2.93; *p* = 0.023; *d* = 1.19), characterized by an increase in volume in the INT and a decrease in the CON (Fig. 3a). No significant differences were observed for ΔT4T0 or ΔT2T0, respectively. Between-group comparisons for each time point separately (T0m, T2m, T4m, or T6m) revealed no significant differences.

### Structural data—hippocampal subfields

The following descriptions focus on hippocampal subfields where significant (or trend-level significant) group × time interactions indicated training intervention-related effects. The complete results of the statistical analyses are listed in Supplementary Table 2.

Right CA4 [body]: A significant interaction effect (*F*(3,73.00) = 3.42; *p* = 0.022), but no main effects (group: *F*(1,22.00) = 0.1, *p* = 0.830; time: *F*(3,73.00) = 0.6, *p* = 0.606) were observed. Post hoc analyses revealed no further effects (Fig. 3b).

Right parasubiculum: A significant interaction effect (*F*(3,73.01) = 2.99; *p* = 0.037) and main effect of group (*F*(1,22.01) = 5,75; *p* = 0.025) were observed, but no main effect of time (*F*(3,73.01) = 0.3, *p* = 0.820). Post hoc analyses showed no further effects (Fig. 3c).

Right presubiculum [head]: A significant interaction (*F*(3,73.00) = 3.89; *p* = 0.012) and main effect of group (*F*(1,22.00) = 5.12; *p* = 0.033), but no main effect of time (*F*(3, 73.01) = 1.0, *p* = 0.391) was observed. Between-group comparisons revealed a significant difference for the change in volume ΔT4T0 (*t*(25) = −3.23; *p* = 0.010; *d* = −1.29) but not for ΔT2T0 or ΔT6T0 (Fig. 3d). Between-group comparisons for each time point separately (T0m, T2m, T4m, or T6m) did not reveal significant differences. Post hoc analyses within groups between time points also did not reveal any further significant effects.

**Fig. 4:**
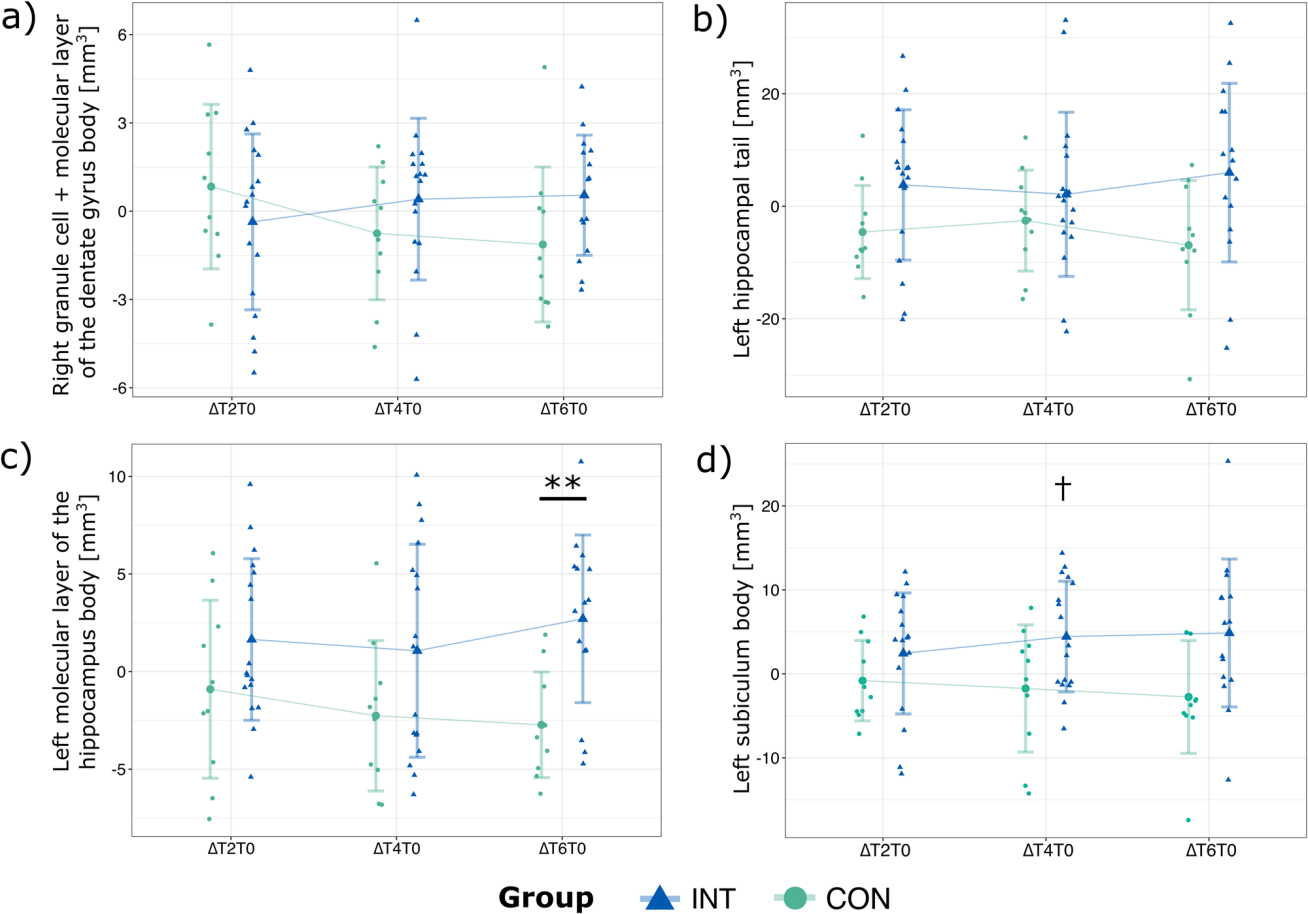
Hippocampal subfields with trends in the time-by-group interaction. **a** Right granule cell and molecular layer of the dentate gyrus body; **b** left hippocampal tail; **c** left molecular layer of the hippocampus body, and **d** left subiculum body. Presented are the deltas between T0m and T2m (ΔT2T0), T0m and T4m (ΔT4T0) as well as T0m and T6m (ΔT6T0) for both groups, along with means and standard deviations (as error bars). ** indicates between-group significances at *p* < 0.01, † indicates within-group significance at *p* < 0.05. Data from *N* = 17 intervention (INT) and *N* = 10 control (CON) participants.

Right granule cell and molecular layer of the dentate gyrus [body]: Analysis revealed a trend for an interaction (*F*(3,73.00) = 2.41; *p* = 0.074), but no main effects (group: *F*(1,21.997) = 0.3, *p* = 0.608; time: *F*(3,73.003) = 0.5, *p* = 0.693). Post hoc analyses revealed no further effects (Fig. 4a).

Left hippocampal tail: Analysis revealed a trend for an interaction (*F*(3,73.01) = 2.19; *p* = 0.097), but no main effects (group: *F*(1,22.01) = 0.9, *p* = 0.366; time: *F*(3,73.01) = 0.0, *p* = 0.996). Post hoc analyses revealed no further effects (Fig. 4b).

Left molecular layer [body]: Analysis revealed a trend for an interaction (*F*(3,73.01) = 2.39; *p* = 0.075), but no main effects (group: *F*(1,22.00) = 1.4, *p* = 0.245; time: *F*(3,73.008) = 0.4, *p* = 0.782). Post hoc analyses within group revealed a trend increase between the time points T0m and T6m in the INT (*t*(14) = −2.44; *p* = 0.086; *d* = −0.63) and a significant decrease between T0m and T6m in the CON group (*t*(9) = 3.19; *p* = 0.033; *d* = −1.01) (Fig. 4c). Importantly, between-group comparisons showed a significant differential change in volume (increase in INT and decrease in CON) for ΔT6T0 (*t*(23) = 3.54; *p* = 0.005; *d* = 1.45) (Fig. 4c). No significant differences were observed for ΔT2T0 or ΔT4T0. Between-group comparisons for each time point separately (T0m, T2m, T4m, or T6m) did not reveal any significant differences.

Left subiculum [body]: Analysis revealed a trend for the interaction (*F*(3,73.01) = 2.41; *p* = 0.073), but no further main effects (group: *F*(1,22.003) = 2.1, *p* = 0.164; time: *F*(3,73.010) = 0.3, *p* = 0.832). Post hoc analyses within group revealed a significant increase between T0m and T4m in INT participants (*t*(16) = −2.78; *p* = 0.041; *d* = −0.67), but no effects between T0m and Tm2 or T0m and T6m (Fig. 4d). Within CON group, no significant effects were observed. Between-group comparisons for changes in volumes (ΔT2T0, ΔT4T0, and ΔT6T0) also revealed no significant effects. There was only a trend for change in volume between groups (increase in INT and decrease in CON) for ΔT6T0 (*t*(23) = 2.32; *p* = 0.089; *d* = 0.95). Between-group comparisons for each time point separately (T0m, T2m, T4m, or T6m) did not reveal any significant differences.

Entorhinal cortex: An exploratory analysis for the left and right entorhinal cortex found no evidence for main effects of group or time, or time-by-group interactions (Supplementary Table 2).

### Correlations between hippocampal volumes, fitness parameters, and spatial memory performance

**Fig. 5:**
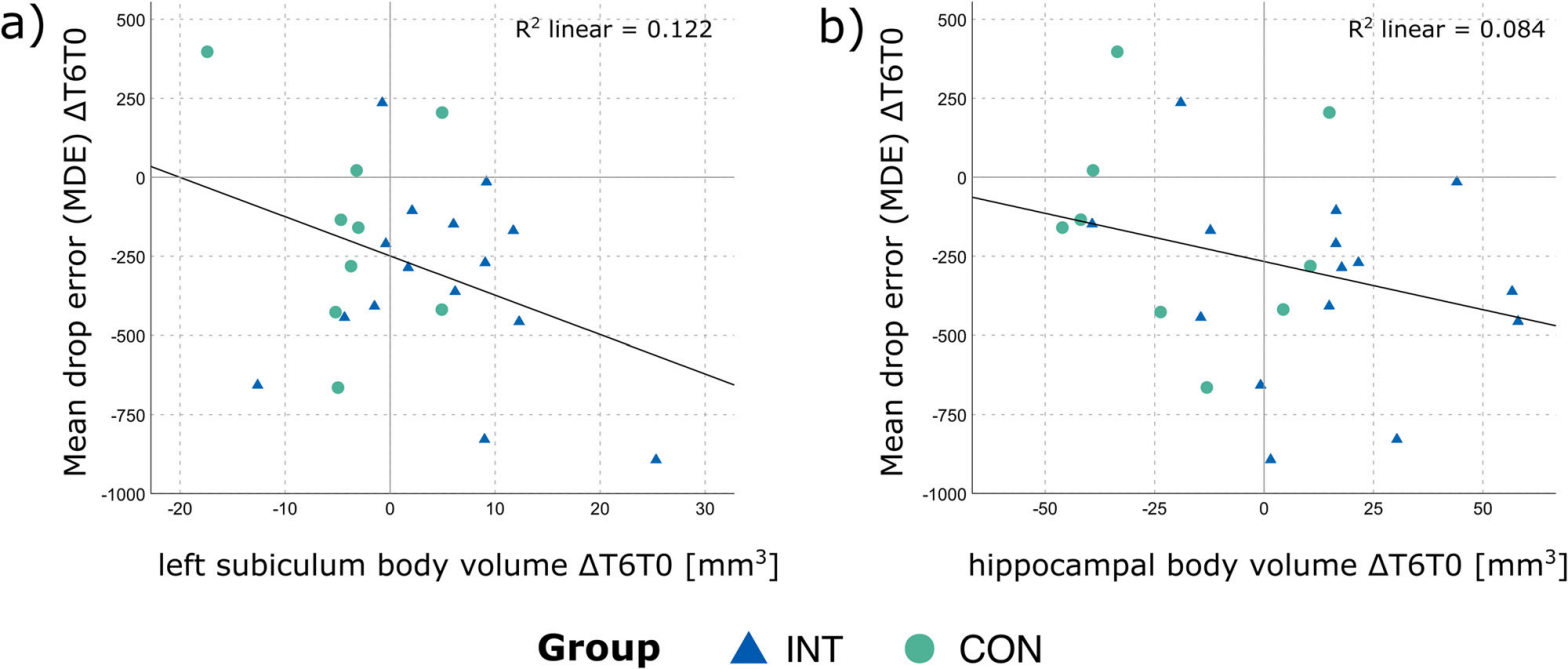
Associations between changes in hippocampal subarea volume and spatial memory precision. Correlations between the changes in Mean Drop Error from baseline to 6-months follow-up (MDE(ΔT6T0)) and the complementary volume changes of the **a**) left subiculum body (ΔT6T0) (*r* = −0.35, significant) and **b**) hippocampal body (ΔT6T0) (*r* = −0.29, trend only). The scatterplots present correlations across groups, i.e., for intervention (INT) and control (CON) participants combined. Data from *N* = 15 INT and *N* = 9 CON participants.

Analyses of both groups combined revealed a significant negative correlation between the MDE and left subiculum body volume (*r* = −0.350, *p* = 0.047), showing that a decrease in MDE goes along with an increase in left subiculum body volume (Fig. 5a). While subgroup analyses showed similar small to moderate negative correlations, these associations were weaker, and missed significance in both groups (INT: *r* = −0.242, *p* = 0.192; CON: *r* = −0.322, *p* = 0.199). Further, an analogous trend was observed for the (negative) correlation between the MDE and the hippocampal body volume (*r* = −0.290, *p* = 0.085) (Fig. 5b). Subgroup analyses showed smaller correlations, which again failed to reach significance (INT: *r* = −0.187, *p* = 0.252; CON: *r* = −0.18, *p* = 0.322).

Meanwhile, we found no evidence for significant positive correlations between relVO_2max_ and volume increases in global hippocampal, subregional or subfield analyses.

## Discussion

This exploratory randomized aerobic exercise intervention over a 6-month period in untrained young adults extends previous evidence that regular physical activity induces changes in fitness, subregional hippocampal plasticity, and spatial learning in humans. As expected, significant increases in fitness were observed in the INT, but not in the CON group, where a slight decrease was seen. Overall improvements in spatial learning in a virtual arena paradigm were found, as indexed by reductions in MDE compared to baseline, and were more robust for the INT than in the CON group (though without significant differences when comparing both groups directly). Consistent with earlier studies in young adults, no evidence was found for training-related changes of global hippocampal volume, as indicated by condition-by-time interactions, but more circumscribed changes in specific subregions. There was a significant interaction for the hippocampal body region, reflected by a trend increase in volume in the INT and decrease in the CON group. At the subfield level, an analogous pattern was observed for the right CA4 body. Similar interactions were found in body regions of the granule cell and molecular layer of the dentate gyrus, left molecular layer, left subiculum, and left hippocampal tail, albeit at trend level only. On the other hand, inverse significant interactions characterized by a trend for volume decreases in the INT and increases in the CON group, respectively, emerged in the right parasubiculum and anterior right presubiculum. Interestingly, correlational analysis focusing on the differences (deltas) between T0m and T6m revealed that MDE reductions, indexing spatial memory improvements over time, correlated with global hippocampal body volume increases as well as with, at the subregional level, volume increases in the left subiculum.

Fitness improvements, demonstrated as relVO_2max_ increases, were found in INT participants only, largely confirming the results of other randomized training interventions in similar age groups of 2-month^60,61^ to 6-month^62^ durations, or meta-analyses of exercise interventions in young adults aged <40 years^63^. Findings indicate that the 6-month aerobic exercise intervention was effective in improving fitness levels, hence, rendering the intervention suitable for subsequent differential statistical analyses of imaging and behavioral changes between time points and groups, as well as their inter-relationships in correlational analyses.

Turning to the structural MRI analyses, whole hippocampal volume showed no significant training-related changes, consistent with previous studies^16^. A meta-analysis from *N* = 23 interventional studies reported a positive effect of exercise on total hippocampal volume in older samples (>65 years), and preferentially in interventions that lasted >24 weeks and <150 min exercise/ week^6^. Hippocampal volume in elderly may be especially sensitive to the positive influence of physical activity because it counteracts ongoing aging-related changes, i.e., serves hippocampal maintenance^10,64^. This anticipated lack of global volume changes in our young adult cohort supported the subregional analysis approach. In fact, we did find evidence for differential changes in the hippocampal body region: Between-group comparisons revealed a significant difference in the pre- (T0) to post- (T6) intervention changes that was driven by volume increase in INT and decrease in CON participants. This regional pattern is interesting, given that previous studies mainly suggested exercise-induced volume changes in anterior (head) subregions: While age-related differences could explain the variation from findings in elderly cohorts^9,11^, an RCT in young- to middle-aged adults^22^ also found a positive effect of a 6-weeks exercise training on anterior (but not whole) hippocampal volume that returned to baseline after a 6-weeks follow-up without training. Another study^23^ indicated positive effects of a 12-week exercise intervention in the left anterior hippocampus. Meanwhile, there is a contrary finding from a voxel-based analysis (i.e., volume decrease in the anterior right hippocampus of the training intervention) after a 6-weeks exercise program^18^. Based on the limited available evidence, it is difficult to evaluate how much the observed heterogeneity relates to methodological factors (e.g., training content and duration).

Considering specific hippocampal subfields, significant training-related changes were detected in the right CA4 body. Anatomically, the multiform layer of the dentate gyrus merges without sharp demarcation into the CA4 region (hilus) of the cornu ammonis^65^ and other human imaging studies have thus reported on one merged dentate & CA4 region (dentate gyrus/CA4)^66,67^. Using slightly different segmentation schemes, recent studies including young adults provide further evidence for training-induced volume increases in dentate gyrus/CA3^23^ and CA4/dentate gyrus subregions^16^, although preferentially in the left hemisphere. Complementary observations from cross-sectional studies also indicate positive associations of CA4 volume with physical fitness levels^32^. In the rodent hippocampus, physical activity has been shown to preferentially facilitate neurogenesis in the dentate gyrus, and its granule cell and molecular layer more specifically^24–26,54,68,69^. In this line, Pereira et al.^29^ compared interventional observations in rodents and young to middle-aged humans: aerobic exercise was found to have a primary effect on cerebral blood volume, an imaging correlate of neurogenesis, in the dentate gyrus of both, mice and men. However, this human 12-week exercise intervention included only a small sample of 11 subjects and had no control group^29^. While the present study found no effects in the dentate gyrus proper, and only a trend-level interaction for the body region of the granule cell and molecular layer of the right dentate gyrus, the latter finding is difficult to interpret, since it was mainly characterized by a trend for volume decrease in the CON group, with no systematic change in the INT group.

Although the animal literature makes the dentate gyrus a promising candidate for exercise-induced plasticity also in the human hippocampus, work by Sorrells et al.^70^ provided evidence that the subgranular zone of the dentate gyrus, contrary to what is well-established in the adult mammalian brain, does not seem to be the major site of neurogenesis in human adults. Interestingly, Nogueira et al.^71^ discuss that neurogenesis in the adult human hippocampal formation may shift from the subgranular zone to the subiculum and the molecular layers of the hippocampus proper^72^. Therefore, it is interesting that the current study found preliminary evidence for exercise-induced volume increases in the left subiculum and the molecular layer. While not regularly observed in exercise-related studies, there is some cross-sectional evidence for a positive relationship between cardiorespiratory fitness and subicular volume in elderly women^73^.

The present analyses also found trend-level evidence for time-by-group interactions in additional subfields, including the left hippocampal tail. Meanwhile, there were also two subfields, the right parasubiculum and right head of the presubiculum, which showed a reversed trend, i.e., smaller volumes for the INT group over time. Given that there is no previous literature showing exercise-induced volume changes in these subregions, these findings are difficult to interpret. In general, it was already noted that some studies in young adults made counterintuitive observations of regional volume reductions after exercise interventions^18^ (see also^14^) which could indicate more complex patterns of subregional structural plasticity that need further systematic exploration, although technical factors may also play a role. These aspects also need to be taken into account when considering the observation that the time-by-group interactions were often driven by both an increasing trend in the training group and a decreasing trend in the control group. Similar patterns were also observed in an earlier study^16^. Although there is preliminary evidence from developmental studies that hippocampal^74^, and also hippocampal subfield volumes^75,76^ may already start to decline in the early 20 s (or even earlier), the limited observation duration (6 months) seems too short to detect such changes reliably on the intraindividual level.

The behavioral outcome of this study, namely, the reduction in MDE as an indicator of improved spatial memory accuracy in the virtual arena was generally shown to improve over time: Exploratory post hoc analyses showed that they were only significant in the INT (reduction compared to baseline), but not in the CON group, even though nominal effect sizes showed that some improvement was also present in this smaller group. Thus, INT participants became significantly more accurate over time in an established spatial learning paradigm^55,56,77,78^. While the influence of practice effects cannot be discarded, as also the CON showed some (nonsignificant) improvement, the effect sizes for the INT were higher, suggesting at least subtle improvements due to exercise. An effect of regular physical activity on spatial learning is supported by previous cross-sectional observations that higher cardiorespiratory fitness was positively linked with mnemonic precision in 2D^45^ and 3D spatial memory task contexts^44,53^. On the other hand, a recent longitudinal study^79^ involving 20 young adults found no direct evidence for improved spatial learning after a 12 weeks mild-intensity aerobic exercise intervention: The intervention neither affected relVO_2max_, nor spatial learning in virtual maze tasks. This negative behavioral outcome, in comparison to our study, might be related to the shorter length and lower intensity of the exercise intervention^79^.

As it comes to possible morphological underpinnings of improved spatial learning after regular exercise training, the reduction in MDE over time was correlated across groups with volume increases in two of the subregions that showed training-related effects, the body part of the left subiculum and, marginally, the hippocampal body (which included the respective subicular subfield). Subgroup analyses showed similar negative trends in both groups, although these effects failed to reach significance: In general, given that the correlation patterns were not specific for the INT group, one has to be careful with inferring a causal relationship between training, structural changes, and spatial memory performance. While hippocampal specialization has long been discussed in the context of an anterior–posterior dichotomy, recent data suggest that at least a tripartite functional scheme including the body region may be more appropriate^20^. There is some evidence that the hippocampal body region is implicated in spatial memory, and that relationships between hippocampal body volume and visuospatial memory performance are not restricted to elderly or clinical populations, but can also be observed in young and middle-aged adults^21^. A specific role of the subiculum in spatial learning is suggested^80^ by lesion studies^81,82^ and cell recordings showing that subicular cells code the animal’s direction during open field exploration, which is not the case for hippocampal place cells^83,84^. However, subicular volume was not regularly associated with task performance in this sort of paradigms in human studies, or visuospatial memory more generally^21^. In fact, previous human studies utilizing tasks for spatial mnemonic discrimination, or spatial navigation, tended to draw relationships with CA1^31,51,52^ or CA3/dentate gyrus integrity^46^. Meanwhile, if changes in cardiorespiratory fitness should modulate subicular volume, such complex task modalities should provide the most sensitive behavioral measure, also given earlier findings in elderly that cardiorespiratory fitness was linked with better performance in a spatial route disambiguation task^44,73^. Although the present and previous studies do not yet provide a coherent picture, they provide evidence that associations between hippocampal structural plasticity and changes in hippocampus-dependent memory function cannot only be observed in aging or early development, but across the whole lifespan.

While strengths include the randomized study design in combination with multiple longitudinal assessments of hippocampal volume changes (instead of simple pre-post comparisons) and behavioral assessment of a complex spatial memory task, this study is not without limitations. Despite some evidence in favor of relative volume increases for the INT group, no linear correlations between fitness gains and volume changes were observed, which would have provided the strongest support for exercise-related changes in hippocampal structure. The hippocampal subfield atlas introduced in FreeSurfer 6^85^ was utilized, which means that the subfield definitions are not completely consistent with those in some abovementioned studies (which used earlier FreeSurfer implementations, or other segmentation schemes). There have been general reservations regarding the reliability of such fully automated segmentation pipelines, although studies using the most recent implementations provide more favorable results^86^. The study was conducted in a mainly European sample, which may limit the generalizability to other ethnic backgrounds. The major limitation that we have to acknowledge is the small number in particular of CON participants finalizing the study which negatively impacted the statistical power of the overall study design, and minimized chances to find significant changes. This limitation is partially reduced by the longitudinal follow-up with three consecutive MRI sessions which confirmed stable results over time in the CON, and very different evolution in the INT. We cannot exclude the risk of false positive findings due to the multitude of performed tests. While analyses for the basic background characteristics indicate no systematic drop-out of participants, we cannot completely exclude selective attrition effects that may overestimate the observed effects, and limit generalizability. As several drop-outs occurred early, it was not feasible to test this directly in a systematic intention-to-treat analysis, due to missing follow-ups. We decided to use a passive control condition, as was discussed in further detail in previous resting-state fMRI^57^ and perfusion studies^58^ from this cohort: While advantages and disadvantages of different types of control conditions have been discussed in the literature, potential bias should not have a major impact on structural data, as in the current study.

In conclusion, this RCT with multiple imaging time-points over a 6-month period in initially untrained young adults identified differential changes in INT, when compared to CON participants. Findings indicate that hippocampal plasticity in young sedentary adults can be induced by regular physical activity, and while there is no evidence for global changes in hippocampal volume (as sometimes observed in elderly), there were regional changes that were partially consistent with animal findings. Meanwhile, spatial learning was particularly linked with local volume increases of the hippocampal body and the subiculum. Because studies of physical activity in young adulthood are generally underrepresented, further interventions should be conducted to elucidate the changes in brain plasticity at this early stage of life that may maximally mediate the long-term benefits of physical activity. Future studies will need larger samples of individuals, ideally over the course of a longer-term intervention (>6 months), to examine the stability of the effects of regular exercise on hippocampal integrity, and incorporate behavioral measures (including functional MRI paradigms) of complex spatial learning abilities. If hippocampal plasticity is not only limited to elderly (serving maintenance against aging-related or neurodegenerative decline), but also present in young adults, this would not only be interesting for clinical populations in this age range but probably also for sedentary individuals who may not only profit from the general health benefits of cardiovascular fitness but also from improved cognitive functioning, especially in hippocampus-related complex memory domains.

## Methods

The present sub-study is part of the “RUNSTUD” study, from which data on other research questions have already been published^57,58,87^. RUNSTUD was a randomized 6-month exercise intervention study in young, healthy, and sedentary subjects which included the acquisition of performance diagnostics, MRI, neuropsychological tests, pain measurements, and blood sampling for epigenetics. The study is registered in the German Register of Clinical Studies (DRKS00021460). A detailed description of the study design was published in Maurer et al.^57^.

### Participants

Healthy sedentary women and men, aged 18–35 years, with no prior history as competitive athletes and/or regular physical exercise training in the last 2 years preceding the study were recruited through social media and via flyers at the local university. Exclusion criteria were psychiatric, neurologic, or cardiovascular diseases (current or in the past). Before each MRI session, participants were informed and screened for contraindications. Female participants were additionally tested for pregnancy.

Written informed consent was obtained after participants were notified about the study purpose, all tests, and potential risks. The study was approved by the Ethics Committee at the Medical Faculty of the Rheinische Friedrich–Wilhelms-Universität Bonn (no. 370/15) and conducted according to national legislation and the Declaration of Helsinki. All ethical regulations relevant to human research participants were followed.

### Experimental procedure

At study inclusion, demographic characteristics (age, educational level, and handedness) and psychiatric questionnaires (such as the Beck Depression Inventory (BDI-I^88^), the trait-anxiety scale of the State-Trait-Anxiety Inventory (STAI^89^), and the Mini International Neuropsychiatric Interview (M.I.N.I., German version 5.0.0^90^)) were obtained from all subjects. In addition, a questionnaire about substance consumption and the Fagerström Test for Nicotine Dependence (FTND^91^) were gathered. To rule out serious pre-existing health conditions, a medical health check was performed subsequent to performance diagnostics and trainings. Thereafter, participants were randomized in one of two groups: the INT group or the passive CON group. The sequential list randomization method was used to assign subjects in a 2:1 ratio (INT:CON) since higher dropout rates in the INT group were expected.

At baseline (T0m) and every 2 months during the study (after two (T2m), four (T4m), and 6 months (T6m)), subjects underwent performance diagnostics (graded exercise test) to determine individual fitness, a 3 Tesla MRI examination, and a spatial object memory task. The INT group received extensive interval training (3 training sessions per week of 25–45 min duration) tailored to their individual fitness levels. The CON group was told not to deviate from their routines. For further details on performance diagnostics and exercise intervention see Maurer et al.^57^. As a fitness outcome, we used the maximum oxygen consumption relative to body weight (relVO_2max_), which was defined as the highest 30 s moving average of VO_2_ divided by body mass (ml min^−1^ kg^−^^1^).

### Spatial object memory task

Participants performed a computerized spatial object memory task, which was adapted from multiple previous studies^55,56,77,78^. These previous studies established that this task has a high validity to investigate spatial memory in humans and that it resembles various spatial navigation and spatial memory tasks in rodents such as the Morris Water Maze task^92^. They demonstrated, amongst others, that the human entorhinal cortex shows a macroscopic functional magnetic resonance imaging (fMRI) signal that may result from the activity of grid cells^93^ and that single neurons in the human medial temporal lobe encode directions and distances during virtual spatial navigation^55^. Previous studies with similar virtual reality paradigms showed that they correlate strongly with real-world spatial memory performance, further supporting the validity of this task for assessing spatial memory^94^.

The paradigm was developed using Unreal Engine 2 (Epic Games, Cary, NC, USA), and is illustrated in Fig. 1. Participants were located in a virtual arena that comprised a grassy plain which was surrounded by a cylindrical cliff. The background scenery included a large and a small mountain, clouds, and the sun: These distal landmarks were rendered at infinity and remained stationary throughout the task. There were no intramaze landmarks. Subjects were able to navigate through the virtual arena by using the arrow keys of a keyboard.

At the beginning of the task, subjects learned the locations of eight everyday objects (randomly drawn from a total number of 12 potential objects: eggplant, baby bottle, briefcase, globe, bucket, rubber duck, barrel, stapler, agenda, vase, alarm clock, basketball) by collecting them from different locations in the arena (“initial learning phase”; total duration 2–3 min). Afterwards, subjects completed 160 trials (20 trials per object in random order), each starting with an inter-trial interval of 3–5 s duration (uniformly distributed). Then, one of the 8 objects (“cue”; duration of 2 s) was presented at the top of the screen. During the subsequent retrieval period (“retrieval”; self-paced), subjects navigated to the remembered object location and indicated their arrival with a button press. Next, subjects received feedback on the accuracy of their response using one of 5 different emoticons (“feedback”; happy to sad; duration of 1.5 s). The retrieved object then appeared in its correct location, and subjects had to navigate there to collect it, to further improve their associative object-location memories (“re-encoding”; self-paced). Because the task was self-paced, total duration depended on how fast the subjects completed the 160 trials.

Every 0.1 s, the participant’s position in the arena was logged, which allowed to extract movement periods, speed, and direction. Response accuracy was measured as the Euclidean distance between the response location and the correct location (“drop error”). To quantify subjects’ spatial memory performance, a mean drop error (MDE) over all 160 trials was calculated by averaging across all raw drop-error values (without any further transformations), which served as a dependent variable for statistical analysis. The lower the MDE, the better the subjects’ spatial memory performance.

To assess the reliability of the computerized spatial object memory task, we estimated the MDE for each session and computed Cronbach’s alpha across different sessions. We found that Cronbach’s alpha was 0.958, indicating excellent reliability of assessing spatial memory performance with this task.

### MRI acquisition

MRI scans were performed on a 3 Tesla Siemens Magnetom Skyra System with a 32-channel head coil. Anatomical T1 weighted (T1w) images were obtained with an in-house developed MP-RAGE sequence with 1 × 3_z1_ CAIPIRINHA and elliptical sampling^95^: sagittal slice orientation, voxel size = 1 × 1 × 1 mm, field-of-view = 192 × 192 × 144 mm, TR = 2.5 s, TI = 1.1 s, TE = 5 ms, flip angle = 7°, total scan duration: 2 min 53 s.

### Hippocampus segmentation

The structural MR images were automatically segmented using the longitudinal stream^96^ provided in FreeSurfer 6.0 (http://surfer.nmr.mgh.harvard.edu/). By means of robust, inverse consistent registration, this workflow generates an unbiased within-subject template space and image^97^. Based on common information from this within-subject template, several of the processing steps of the default FreeSurfer pipeline^98^ for cross-sectional data (e.g., skull stripping, Talairach transformation, atlas registration, spherical surface mapping and parcellation) are initialized, which increases reliability and statistical power of the longitudinal stream significantly^96^.

Hippocampal subfield segmentation utilized the hippocampal subfields and amygdala nuclei atlas^85^ in FreeSurfer 7.0. Technical details regarding the procedure and segmented hippocampal subfield can be found online (https://surfer.nmr.mgh.harvard.edu/fswiki/HippocampalSubfieldsAndNucleiOfAmygdala). Volume measurements were obtained from 12 subfields, each for the left and right hemispheres, and some of them were further divided into head and body divisions. Subfields were: (i) parasubiculum, (ii) presubiculum [head and body], (iii) subiculum [head and body], (iv) CA1 [head and body], (v) CA3 [head and body] which also includes CA2, (vi) CA4 [head and body], (vii) granule cell and molecular layer of the dentate gyrus [granule cells-molecular layer-dentate gyrus, head and body], (viii) molecular layer [head and body], (ix) hippocampal amygdaloid transition area, (x) fimbria, (xi) hippocampal tail, and (xii) hippocampal fissure. Segmentation resulted in 19 subdivisions (including head/body divisions) and a total of 38 subdivisions across hemispheres. For comparison with previous investigations, the subfield volumes were also combined to calculate a whole hippocampus measure, and, following suggestions that exercise effects may vary along the longitudinal hippocampal axis^9,11,18,22,23^, created composites for head (including left and right parasubiculum, presubiculum [head], subiculum [head], CA1 [head], CA3 [head], CA4 [head], granule cell and molecular layer of dentate gyrus [head], molecular layer of the hippocampus [head], and hippocampal amygdaloid transition area), body (including left and right presubiculum [body], subiculum [body], CA1 [body], CA3 [body], CA4 [body], granule cell and molecular layer of dentate gyrus [body], molecular layer of the hippocampus [body], and fimbria) and tail (left and right hippocampal tail).

Due to previous studies with the behavioral task that indicated a central role of the entorhinal cortex^56^, we also analyzed the left and right entorhinal volumes for exploratory purposes.

Finally, the pipeline included the estimation of total intracranial volume (eTIV) for each subject^99^ as a covariate for later statistical analyses.

The ENIGMA quality control protocol (http://enigma.ini.usc.edu/) was used for visual quality control of the automatic hippocampus segmentation^100^.

### Statistics and reproducibility

Statistical analyses were conducted using IBM SPSS Statistics version 27 (IBM Corp., Armonk, New York). To test for baseline differences between groups, participants´ characteristics were analyzed using independent t-tests for interval data, and *χ*^2^ tests for frequency data.

Analyses of longitudinal physiological (relVO_2max_), behavioral (Spatial Object Memory Task), and structural MRI data (hippocampal subfields) were performed using linear mixed effects (LME) models. Covariates age and sex (as fixed effects) and random intercepts were added to account for random individual level effects. Additionally, eTIV was added as a covariate of no interest in structural data analyses to account for variations in individual brain size.

Assumptions for applying the linear mixed effects model (linearity and normality) were tested by performing Shapiro-Wilk tests and visual checks of the Q–Q plots of the model residuals for normality and by visually checking the plots between model residuals vs the predictor for linearity. Further, we checked for outliers by checking the distribution of residuals. All hippocampal subfield and MDE residuals showed approximately normal distributions (except for the hippocampal subfield “right CA3 [body]”). Further, all plots of the hippocampal subfield and the MDE predictors and their LME model residuals looked random, in line with the assumption of linearity. The investigation of outliers revealed no extreme outliers. Therefore, we included all subjects’ data for final analysis.

In case of significant effects (time, group, or time-by-group interaction), post hoc tests between time points within each group were conducted. Between-group comparisons were performed for each time point separately but also for the change in volume over time (delta T2m minus T0m (ΔT2T0), delta T4m minus T0m (ΔT4T0), and delta T6m minus T0m (ΔT6T0)). *p*-values were adjusted using Bonferroni correction. Results of post hoc tests were considered significant when *p* < 0.05 and are reported with Cohen’s *d* effect size^101^.

The subfields hippocampal amygdaloid transition area and hippocampal fissure were not of primary interest in this study and therefore are reported in Supplementary Note 1 and Supplementary Fig. 1.

#### Correlation analyses

Correlation analyses were performed between the change in relVO_2max_ and behavioral changes in the Spatial Object Memory Task (MDE) and changes in the hippocampal subfield volumes. Therefore, differences between T0m and T6m were calculated by subtracting baseline values from the follow-up assessment. Due to the small sample sizes, we only correlated the values of both groups (INT and CON) together. Positive associations between increased subregional volume and increased relVO_2max_ or decreased MDE, respectively, were considered significant (*p* < 0.05, one-tailed).

### Reporting summary

Further information on research design is available in the Nature Portfolio Reporting Summary linked to this article.

### Supplementary information

Supplementary information

Description of Additional Supplementary Files

Supplementary Data 1

Reporting Summary

## Data Availability

The datasets generated during and/or analyzed during the current study are not publicly available due to containing information that could compromise research participant privacy/consent. Data can only be made available by the corresponding author (H.B.) after contacting participating volunteers and obtaining their consent to submit data in pseudonymized form (as per ethics’ approval). Depending on the decision of the volunteers, this may result in smaller samples. The data used to make the plots in Figs. 2–5 can be found in Supplementary Data 1.

## Code availability

The spatial object memory task was adapted from previous studies (Doeller et al., 2008: 10.1073/pnas.0711433105; Kunz et al, 2015: 10.1126/science.aac8128), and developed using Unreal Engine 2 (Epic Games, Cary, NC, USA). Custom MATLAB code generated during this study for behavioral data analysis of the Spatial Object Memory Task is available at https://github.com/NeuroLuke/BoeckerCommunicationsBiology2024. Structural MRI data were preprocessed using the FreeSurfer 6.0 and 7.0 standard workflows for longitudinal and hippocampal subfield segmentation, respectively. Demographic, behavioral, physiological and volumetric data were analyzed using default procedures provided in SPSS 27.0 (IBM Corp, Armonk, NY).
