# Supplementary material for: Hippocampal subfield plasticity is associated with improved spatial memory
Source: Commun Biol. 2024 Mar 5;7:271. doi: 10.1038/s42003-024-05949-5 (PMC10914736; doi:10.1038/s42003-024-05949-5)
Supplement: Supplementary file 1 — Supplementary information [file 42003_2024_5949_MOESM1_ESM.pdf]

Supplementary information for:

Hippocampal subfield plasticity is associated with improved spatial memory

Authors:

Henning Boecker[1,2], Marcel Daamen[2], Lukas Kunz[3], Melanie Geiß[1], Moritz Müller[1], Thomas Neuss[1], Leonie Henschel[2], Rüdiger Stirnberg[2], Neeraj Upadhyay[1,2], Lukas Scheef[1], Jason A. Martin[1], Tony Stöcker[2], Alexander Radbruch[4], Ulrike Attenberger[5], Nikolai Axmacher[6], Angelika Maurer[1,2]

Affiliations:

[1] Clinical Functional Imaging Lab, Department of Diagnostic and Interventional Radiology, University Hospital Bonn, Venusberg-Campus 1, 53127 Bonn, Germany

[2] German Center for Neurodegenerative Diseases, Venusberg-Campus 1/99, 53127 Bonn, Germany

[3] Department of Epileptology, University of Bonn Medical Centre, Bonn, Germany

[4] Department of Neuroradiology, University Hospital Bonn, Venusberg-Campus 1, 53127 Bonn, Germany

[5] Department of Diagnostic and Interventional Radiology, University Hospital Bonn, Venusberg-Campus 1, 53127 Bonn, Germany

[6] Department of Neuropsychology, Faculty of Psychology, Ruhr University Bochum, Bochum, Germany

Corresponding Author

Prof. Henning Boecker

Group Leader of Clinical Functional Imaging Lab

Department of Diagnostic and Interventional Radiology

University Hospital Bonn

Venusberg-Campus 1, Building 07

D-53127 Bonn

Phone: +49 228 – 287 15980

Email: henning.boecker@ukbonn.de

ORCID: https://orcid.org/0000-0003-2346-0598

Supplementary Table 1: Background characteristics for included cases vs. drop-outs

Variable	Included<br>N=27 (17 INT/10 CON)	Dropouts<br>N=15 (10 INT/5 CON)	Statistics
Sex (m/f)	12/15	8/7	χ^2(1, N= 42) = 0.05, p = 0.827
Age [years]	23.9 ± 4.0	24.5 ± 4.1	t(40)=-0.49, p=0.626, d=-0.16
Height [cm]	174.8 ± 10.8	174.0 ± 8.5	t(40)=0.25, p=0.804, d=0.08
Weight [kg]	70.4 ± 14.5	75.3 ± 17.9	t(40)=-0.95, p=0.346, d=-0.31
BMI [$kg \cdot m^{-2}$]	22.9 ± 3.6	24.9 ± 5.7	t(40)=-1.33, p=0.191, d=-0.44
HR_{max} [bpm]	199 ± 8	197 ± 9	t(39)=0.92, p=0.361, d=0.31
WST IQ	107 ± 9	104 ± 8	t(38)=0.97, p=0.338, d=0.34

Abbreviations: BMI = body mass index; CON = control group; HR_{max} = maximum heart rate in performance diagnostic; INT = intervention group; WST IQ = verbal intelligence quotient derived from German vocabulary test.

Supplementary Table 2: Main effects and interactions for the analyzed hippocampal regions

Region	Fixed effects		
	Group	Time	Time-by-group interaction
Global			
Whole hippocampus	F(1, 22.002) = 4.1, p = .055	F(3, 73.004) = 0.7, p = .533	F(3, 73.004) = 0.1, p = .960
Subregions			
Head	F(1, 22.003) = 5.5, p = .029	F(3, 73.006) = 0.4, p = .764	F(3, 73.006) = 0.9, p = .437
Body	F(1, 22.000) = 1.8, p = .190	F(3, 73.003) = 0.5, p = .679	F(3, 73.003) = 2.9, p = .041
Whole tail	F(1, 22.004) = 1.1, p = .312	F(3, 73.007) = 0.5, p = .680	F(3, 73.007) = 1.3, p = .275
Subfields			
Left parasubiculum	F(1, 21.998) = 0.5, p = .509	F(3, 73.011) = 1.3, p = .292	F(3, 73.011) = 0.4, p = .756
Right parasubiculum	F(1, 22.006) = 5.7, p = .025	F(3, 73.013) = 0.3, p = .820	F(3, 73.013) = 3.0, p = .037
Left presubiculum head	F(1, 22.003) = 0.1, p = .828	F(3, 73.007) = 1.4, p = .255	F(3, 73.007) = 1.2, p = .322
Right presubiculum head	F(1, 21.998) = 5.2, p = .033	F(3, 73.005) = 1.0, p = .391	F(3, 73.005) = 3.9, p = .012
Left presubiculum body	F(1, 22.004) = 2.3, p = .142	F(3, 73.009) = 1.1, p = .350	F(3, 73.009) = 0.5, p = .667
Right presubiculum body	F(1, 21.999) = 0.0, p = 936	F(3, 73.003) = 1.2, p = .159	F(3, 73.003) = 1.9, p = .145
Left subiculum head	F(1, 22.001) = 5.5, p = .029	F(3, 73.008) = 0.6, p = .612	F(3, 73.008) = 0.3, p = .845
Right subiculum head	F(1, 21.983) = 7.8, p = .011	F(3, 72.989) = 1.0, p = .401	F(3, 72.989) = 0.3, p = .793
Left subiculum body	F(1, 22.003) = 2.1, p = .164	F(3, 73.010) = 0.3, p = .832	F(3, 73.010) = 2.4, p = .073
Right subiculum body	F(1, 21.991) = 2.0, p = .174	F(3, 72.996) = 2.4, p = .075	F(3, 72.996) = 1.0, p = .398
Left CA1 head	F(1, 22.005) = 2.1, p = .160	F(3, 73.009) = 0.7, p = .572	F(3, 73.009) = 0.5, p = .680
Right CA1 head	F(1, 22.004) = 5.4, p = .030	F(3, 73.008) = 1.0, p = .403	F(3, 73.008) = 1.0, p = .402
Left CA1 body	F(1, 22.003) = 0.8, p = .379	F(3, 73.006) = 0.8, p = .507	F(3, 73.006) = 1.2, p = .330
Right CA1 body	F(1, 22.001) = 0.5, p = .484	F(3, 73.003) = 0.7, p = .584	F(3, 73.003) = 0.0, p = .990
Left CA3 head	F(1, 21.996) = 0.4, p = .544	F(3, 73.000) = 0.7, p = .588	F(3, 73.000) = 0.5, p = .680
Right CA3 head	F(1, 21.974) = 0.1, p = .755	F(3, 72.980) = 0.3, p = .804	F(3, 72.980) = 0.7, p = .564
Left CA3 body	F(1, 22.001) = 0.4, p = .517	F(3, 73.004) = 0.1, p = .945	F(3, 73.004) = 0.6, p = .634
Right CA3 body	F(1, 21.995) = 0.4, p = .532	F(3, 72.999) = 0.3, p = .805	F(3, 72.999) = 0.9, p = .455
Left CA4 head	F(1, 21.996) = 1.3, p = .273	F(3, 73.004) = 0.2, p = .922	F(3, 73.004) = 0.5, p = .667
Right CA4 body	F(1, 21.999) = 0.1, p = .830	F(3, 73.004) = 0.6, p = .606	F(3, 73.004) = 3.4, p = .002

- Continued -

Supplementary Table 2 (continued)

**Region**	**Fixed effects**		
	**Group**	**Region**	**Group*time interaction**
Subfields			
Left CA4 body	F(1, 22.007) = 0.0, p = .924	F(3, 73.017) = 1.3, p = .276	F(3, 73.017) = 0.2, p = .875
Right CA4 head	F(1, 21.980) = 0.3, p = .583	F(3, 72.986) = 2.0, p = .116	F(3, 72.986) = 0.4, p = .793
Left fimbria	F(1, 22.016) = 4.1, p = .056	F(3, 73.029) = 1.5, p = .213	F(3, 73.029) = 0.3, p = .865
Right fimbria	F(1, 22.022) = 0.3, p = .574	F(3, 73.053) = 1.3, p = .292	F(3, 73.053) = 2.1, p = .103
Left GC ML DG head	F(1, 21.997) = 1.8, p = .197	F(3, 73.002) = 0.3, p = .815	F(3, 73.002) = 0.5, p = .677
Right GC ML DG head	F(1, 21.991) = 0.3, p = .569	F(3, 72.996) = 0.8, p = .489	F(3, 72.996) = 1.4, p = .239
Left GC ML DG body	F(1, 22.008) = 0.0, p = .977	F(3, 73.018) = 1.2, p = .310	F(3, 73.018) = 0.5, p = .705
Right GC ML DG body	F(1, 21.997) = 0.3, p = .608	F(3, 73.003) = 0.5, p = .693	F(3, 73.003) = 2.4, p = .074
Left molecular layer HP head	F(1, 22.003) = 3.2, p = .087	F(3, 73.007) = 0.1, p = .941	F(3, 73.007) = 0.3, p = .838
Right molecular layer HP head	F(1, 22.004) = 3.5, p = .077	F(3, 73.009) = 0.8, p = .505	F(3, 73.009) = 0.4, p = .730
Left molecular layer HP body	F(1, 22.003) = 1.4, p = .245	F(3, 73.008) = 0.4, p = .782	F(3, 73.008) = 2.4, p = .075
Right molecular layer HP body	F(1, 21.996) = 0.3, p = .611	F(3, 72.999) = 2.6, p = .059	F(3, 72.999) = 0.5, p = .676
Left Hippocampus tail	F(1, 22.005) = 0.9, p = .366	F(3, 73.009) = 0.0, p = .996	F(3, 73.009) = 2.2, p = .097
Right Hippocampus tail	F(1, 22.004) = 1.1, p = .317	F(3, 73.008) = 1.6, p = .183	F(3, 73.008) = 0.8, p = .508
Left HATA	F(1, 21.981) = 0.0, p = .869	F(3, 72.990) = 2.7, p = .053	F3, 72.990) = 2.6, p = .058
Right HATA	F(1, 21.992) = 0.2, p = .647	F(3, 73.002) = 2.3, p = .080	F(3, 73.002) = 2.7, p = .051
Left hippocampal fissure	F(1,21.983) = 0.1, p = .731	F(3, 73.005) = 1.5, p = .215	F(3, 73.005) = 2.1, p = .111
Right hippocampal fissure	F(1, 22.006) = 0.2, p = .648	F(3, 73.032) = 2.2, p = .100	F(3, 73.032) = 2.4, p = .071
Exploratory			
Left entorhinal	F(1, 21.928) = 0.0, p = .949	F(3, 72.967) = 0.3, p = .812	F(3, 72.967) = 0.9, p = .462
Right entorhinal	F(1, 21.918) = 3.0, p = .099	F(3, 72.980) = 1.8, p = .153	F(3, 72.980) = 0.7, p = .548

Abbreviations: CA – Cornu ammonis. GC – Granular cells. HATA - Hippocampus-Amygdala-Transition-Area. HP – Hippocampus. ML – Molecular layer.

Supplementary Note 1: Additional results

Right Hippocampus-Amygdala-Transition-Area: Analysis revealed a strong trend for the interaction ($F(3,73.00)=2.71$; $p=0.051$), driven by a decrease in volume in the INT and no change in volume in the CON group from T0m to T6m (Supplementary Figure 1a). No further main effects (group or time) were observed. Post hoc analyses within group revealed a significant decrease from T0m to T2m in the INT ($t(16)=4.27$; $p=0.002$; $d=1.04$) and a trend decrease from T0m to T4m ($t(16)=2.43$; $p=0.082$; $d=0.59$). However, there was no significant difference between T0m and T6m in the INT group. In the CON group, no significant differences between time points was observed. Between-group comparisons for the change in volume (ΔT2T0, ΔT4T0, and ΔT6T0) only revealed a trend for ΔT2T0 ($t(25)=-2.50$; $p=0.058$; $d=-1.00$). Between-group comparisons for each time point separately (T0m, T2m, T4m, or T6m) did not reveal significant differences.

Right hippocampal fissure: Analysis revealed a trend for the interaction ($F(3,73.03)=2.44$; $p=0.071$), driven by an increase in both groups from T0m to T6m (Supplementary Figure 1b). No further main effects (group or time) were observed. Post hoc analyses revealed no further significant effects.

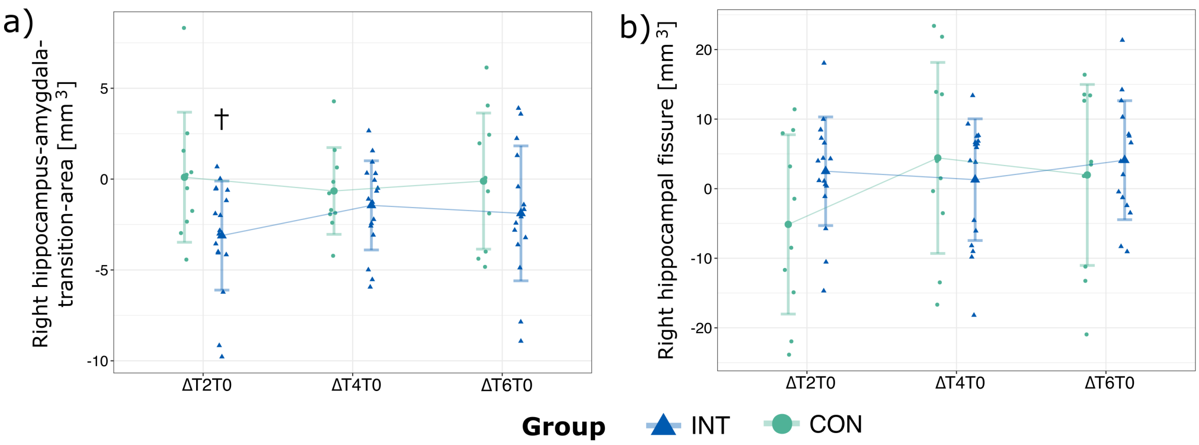

Supplementary Figure 1: Hippocampal subfields with trends in the group x time interaction. a) Right hippocampus-amygdala-transition-area and b) right hippocampal fissure. Presented are the delta values between T0m and T2m (ΔT2T0), T0 and T4 (ΔT4T0) as well as T0m and T6m (ΔT6T0) for both, the intervention group (INT) and the control (CON) group, along with means and standard deviations (shown as error bars). † indicates within-group significance at $p<0.05$. The data used to make the plots can be found in the Supplementary Data 1 file for this article.
