# Supplementary material for: Hippocampal subfield plasticity is associated with improved spatial memory
Source: Commun Biol. 2024 Mar 5;7:271. doi: 10.1038/s42003-024-05949-5 (PMC10914736; doi:10.1038/s42003-024-05949-5)
Supplement: Supplementary file 4 — Reporting Summary [file 42003_2024_5949_MOESM4_ESM.pdf]

Corresponding author(s): Prof. Henning Boecker

Last updated by author(s): Feb 1, 2024

Reporting Summary

Nature Portfolio wishes to improve the reproducibility of the work that we publish. This form provides structure for consistency and transparency in reporting. For further information on Nature Portfolio policies, see our Editorial Policies and the Editorial Policy Checklist.

Statistics

For all statistical analyses, confirm that the following items are present in the figure legend, table legend, main text, or Methods section.

n/a	Confirmed	
☐	☒	The exact sample size (*n*) for each experimental group/condition, given as a discrete number and unit of measurement
☐	☒	A statement on whether measurements were taken from distinct samples or whether the same sample was measured repeatedly
☐	☒	The statistical test(s) used AND whether they are one- or two-sided<br>*Only common tests should be described solely by name; describe more complex techniques in the Methods section.*
☐	☒	A description of all covariates tested
☐	☒	A description of any assumptions or corrections, such as tests of normality and adjustment for multiple comparisons
☐	☒	A full description of the statistical parameters including central tendency (e.g. means) or other basic estimates (e.g. regression coefficient) AND variation (e.g. standard deviation) or associated estimates of uncertainty (e.g. confidence intervals)
☐	☒	For null hypothesis testing, the test statistic (e.g. *F*, *t*, *r*) with confidence intervals, effect sizes, degrees of freedom and *P* value noted<br>*Give P values as exact values whenever suitable.*
☒	☐	For Bayesian analysis, information on the choice of priors and Markov chain Monte Carlo settings
☒	☐	For hierarchical and complex designs, identification of the appropriate level for tests and full reporting of outcomes
☐	☒	Estimates of effect sizes (e.g. Cohen's *d*, Pearson's *r*), indicating how they were calculated

Our web collection on statistics for biologists contains articles on many of the points above.

Software and code

Policy information about availability of computer code

Data collection	The spatial object memory task was adapted from previous studies (Doeller et al., PNAS 2008: https://doi.org/10.1073/pnas.0711433105; Kunz et al, Science 2015: https://doi.org:10.1126/science.aac8128) and developed using Unreal Engine 2 (Epic Games, Cary, NC, USA).
Data analysis	Custom MATLAB code generated during this study for behavioral data analysis of the Spatial Object Memory Task is available at https://github.com/NeuroLuke/BoeckerCommunicationsBiology2024.<br>Imaging data were processed using FreeSurfer 6.0 (for longitudinal registration/ segmentation) and 7.0 (for hippocampal subfield segmentation), using FreeSurfer standard workflows.<br>Demographic, behavioral, physiological and volumetric data were analyzed using default procedures in SPSS 27.0 (IBM Corp, Armonk, NY).

For manuscripts utilizing custom algorithms or software that are central to the research but not yet described in published literature, software must be made available to editors and reviewers. We strongly encourage code deposition in a community repository (e.g. GitHub). See the Nature Portfolio guidelines for submitting code & software for further information.

Data

Policy information about availability of data

All manuscripts must include a data availability statement. This statement should provide the following information, where applicable:

- Accession codes, unique identifiers, or web links for publicly available datasets
- A description of any restrictions on data availability
- For clinical datasets or third party data, please ensure that the statement adheres to our policy

The datasets generated during and/or analysed during the current study are not publicly available due to containing information that could compromise research participant privacy/consent. Data can only be made available by the corresponding author after contacting participating volunteers and obtaining their consent to submit data in pseudonymized form (as per ethics' approval). Depending on the decision of the volunteers, this may result in smaller samples.

Research involving human participants, their data, or biological material

Policy information about studies with human participants or human data. See also policy information about sex, gender (identity/presentation), and sexual orientation and race, ethnicity and racism.

Reporting on sex and gender	Information regarding sex of the participating individuals (males and females, based on self-report) were collected, and used as a covariate in statistical analysis. As we had no a priori hypotheses regarding sex-specific effects, sex was not used as a stratification variable in the randomization process. Due to the limited sample sizes, we did also not perform sex-stratified group analyses.
Reporting on race, ethnicity, or other socially relevant groupings	The study was conducted in a mainly European sample, but this was not subject of the study design and analyses.
Population characteristics	The study examined healthy young adults (age 18-35 years) without prior history as competitive athletes and/or regular physical exercise training in the previous 2 years. Exclusion criteria were psychiatric, neurologic, or cardiovascular diseases (current or in the past). Before each MRI session, participants were informed and screened for contraindications. Female participants were additionally tested for pregnancy before each MR examination.
Recruitment	The study was approved by theVolunteers were recruited through social media and via flyers at the local university. Ethics Committee at the Medical Faculty of the Rheinische Friedrich-Wilhelms-Universität Bonn (no. 370/15) and conducted according to national legislation and the Declaration of Helsinki.
Ethics oversight	The study was approved by the Ethics Committee at the Medical Faculty of the Rheinische Friedrich-Wilhelms-Universität Bonn (no. 370/15) and conducted according to national legislation and the Declaration of Helsinki.

Note that full information on the approval of the study protocol must also be provided in the manuscript.

Field-specific reporting

Please select the one below that is the best fit for your research. If you are not sure, read the appropriate sections before making your selection.

☒ Life sciences ☐ Behavioural & social sciences ☐ Ecological, evolutionary & environmental sciences

For a reference copy of the document with all sections, see nature.com/documents/nr-reporting-summary-flat.pdf

Life sciences study design

All studies must disclose on these points even when the disclosure is negative.

Sample size	This study had a final sample size of N = 27 subjects with N = 17 in the exercise intervention group and N = 10 in the control group. These subjects were scanned four times each at two-month intervals for a total 6-month time-period.
Data exclusions	Detailed descriptions are provided in earlier publications (Upadhyay et al., Frontiers in Aging Neuroscience 2022: https://doi.org/10.3389/fnagi.2022.951022). In brief, N=57 participants were assessed for basic eligibility after giving their informed consent. Of these, N=48 subjects were randomized into the intervention group (INT, N=32) and control group (CON, N=16). Yet, N=6 participants (N=5 INT, N=1 CON) already dropped out during baseline assessments (due to panic attacks in the MR, or loss of interest), leaving N=27 INT and 15 CON who received the allocated treatment.<br>During the intervention phase, another 10 participants dropped out for various reasons, including loss of interest, or injuries caused by private activities, leaving N=21 INT and N=11 CON participants who completed the intervention phase.<br>For the present analyses, N=4 INT and N=1 CON participant had excluded retrospectively, due to the development of depressive symptoms during the intervention, missing 3T MR data, insufficient adherence to the intervention schedule, and (specific for the present analyses) lack of compliance in the Spatial Object Memory Task.
Replication	Due to the limited sample size, all available datasets were used, and no replication was performed in separate cohorts. However, further studies with larger sample sizes are warrented.

Randomization	Subjects were randomly assigned to either the exercise intervention group or the control group. Due to the expected higher dropout rate in the exercise intervention group subjects were assigned in a 2:1 ratio (intervention : control) using the sequential list randomization method.
Blinding	As by nature, subjects could not be blinded with regard to being randomized to the exercise intervention group or the control group.

Reporting for specific materials, systems and methods

We require information from authors about some types of materials, experimental systems and methods used in many studies. Here, indicate whether each material, system or method listed is relevant to your study. If you are not sure if a list item applies to your research, read the appropriate section before selecting a response.

Materials & experimental systems

n/a	Involved in the study	
☒	☐	Antibodies
☒	☐	Eukaryotic cell lines
☒	☐	Palaeontology and archaeology
☒	☐	Animals and other organisms
☒	☐	Clinical data
☒	☐	Dual use research of concern
☒	☐	Plants

Methods

n/a	Involved in the study	
☒	☐	ChIP-seq
☒	☐	Flow cytometry
☐	☒	MRI-based neuroimaging

Plants

Seed stocks	N/A
Novel plant genotypes	N/A
Authentication	N/A

Magnetic resonance imaging

Experimental design

Design type	N/A
Design specifications	N/A
Behavioral performance measures	Participants performed a computerized spatial object memory task outside of the scanner (i.e., no task fMRI) where the position of objects had to be learned and recollected while navigating in a 3D virtual arena. Spatial memory performance was operationalized by the mean drop error, i.e. the Euclidian distance between the response location and the correct object location, averaged over 160 trials per session. Behavioral performance changes were correlated with structural MRI changes

Acquisition

Imaging type(s)	Structural MRI
Field strength	3T
Sequence & imaging parameters	MRI scans were performed at a 3T Siemens Magnetom Skyra System with a 32-channel head-coil. Anatomical T1 weighted (T1w) images were acquired using an in-house developed MP-RAGE sequence with 1x3z1 CAIPIRINHA and elliptical sampling with the following specifications: sagittal slice orientation, voxel size = 1 x 1 x 1 mm, field-of-view = 192 x 192 x 144 mm, TR = 2.5 s, TI = 1.1 s, TE = 5 ms, flip angle = 7°, total scan duration: 2 minutes 53 seconds.
Area of acquisition	Whole brain

Diffusion MRI ☐ Used ☒ Not used

Preprocessing

Preprocessing software	FreeSurfer 6.0 and 7.0 (http://surfer.nmr.mgh.harvard.edu/)
Normalization	Normalization to MNI space for brain parcellation purposes, final volumes analyzed in native space (with correction for estimated total intracranial volume).
Normalization template	Desikan-Killiany
Noise and artifact removal	N4 bias correction
Volume censoring	N/A

Statistical modeling & inference

Model type and settings	linear mixed effects (LME) model
Effect(s) tested	In case of significant effects (time, group, or time by group interaction), post hoc tests between time points within each group were conducted. Between-group comparisons were performed for each time point separately but also for the change in volume over time (Delta T2 minus T0 (DT2T0), DT4T0, and DT6T0).

Specify type of analysis: ☐ Whole brain ☒ ROI-based ☐ Both

Anatomical location(s): Hippocampus

Statistic type for inference (See Eklund et al. 2016)	N/A
Correction	Results of post hoc tests were considered significant when $p < 0.05$ (two-sided, Bonferroni-corrected) and are reported with Cohen´s d as an effect size

Models & analysis

n/a	Involved in the study
☒	☐ Functional and/or effective connectivity
☒	☐ Graph analysis
☒	☐ Multivariate modeling or predictive analysis
